# A False Trail to Follow: Differential Effects of the Facial Feedback Signals From the Upper and Lower Face on the Recognition of Micro-Expressions

**DOI:** 10.3389/fpsyg.2018.02015

**Published:** 2018-10-24

**Authors:** Xuemei Zeng, Qi Wu, Siwei Zhang, Zheying Liu, Qing Zhou, Meishan Zhang

**Affiliations:** Cognition and Human Behavior Key Laboratory of Hunan Province, Department of Psychology, Hunan Normal University, Changsha, China

**Keywords:** micro-expression, facial feedback, amplified, blocked, micro-expression recognition

## Abstract

Micro-expressions, as fleeting facial expressions, are very important for judging people’s true emotions, thus can provide an essential behavioral clue for lie and dangerous demeanor detection. From embodied accounts of cognition, we derived a novel hypothesis that facial feedback from upper and lower facial regions has differential effects on micro-expression recognition. This hypothesis was tested and supported across three studies. Specifically, the results of Study 1 showed that people became better judges of intense micro-expressions with a duration of 450 ms when the facial feedback from upper face was enhanced via a restricting gel. Additional results of Study 2 showed that the recognition accuracy of subtle micro-expressions was significantly impaired under all duration conditions (50, 150, 333, and 450 ms) when facial feedback from lower face was enhanced. In addition, the results of Study 3 also revealed that blocking the facial feedback of lower face, significantly boosted the recognition accuracy of subtle and intense micro-expressions under all duration conditions (150 and 450 ms). Together, these results highlight the role of facial feedback in judging the subtle movements of micro-expressions.

## Introduction

Facial expressions are the most intuitive windows for people to express their emotions and intentions. Accurately recognizing facial expressions of emotion is arguably the most important social task we engage as social creatures (Niedenthal and Brauer, 2012). However, not all emotions are necessarily displayed on the face. As a social creature, we all have the motivation to deceive or manipulate others. To achieve that purpose, in a number of situations, we all need to hide, disguise, and inhibit our true emotions (Ekman, 1971). Researchers have found that, despite our efforts to hide, the true emotions may still leak. These leaked emotions are usually displayed in the form of micro-expressions (Ekman and O’Sullivan, 2006; Ekman, 2009; Porter et al., 2012; Yan et al., 2013). Micro-expression is a fleeting facial expression which lasts no longer than 0.5 s (Frank et al., 2009b; Matsumoto and Hwang, 2011; Yan et al., 2013). It usually occurs in the high-stake situations, especially for people who have something important to gain or lose (Ekman, 2009). Because of its involuntary nature, accurately detecting these micro-expressions has great application potentials for individuals whose profession requires face-to-face interpersonal skills, such as health care workers, psychotherapists, and law enforcement officers (e.g., Russell et al., 2006; Russell et al., 2008; Endres and Laidlaw, 2009; Frank et al., 2009b; Warren et al., 2009; Marsh et al., 2010). In addition, due to the close relationship between micro-expression and deception, recognizing micro-expressions is believed to be one of the most reliable methods to detect lies (e.g., Weinberger, 2010; ten Brinke and Porter, 2012; Frank and Svetieva, 2015).

However, the duration of micro-expression is very short. For a typical micro-expression, usually it only lasts for 40–500 ms (Ekman, 2003; Matsumoto and Hwang, 2011; Yan et al., 2013). It is very difficult for observers to accurately detect and recognize these fleeting facial expressions in social situations (Ekman and Friesen, 1969; Ekman, 2003; Svetieva and Frank, 2016). Conservative tests in the laboratory (by sandwiching the briefly presented image of facial expression between two masking neutral faces) also found that it was difficult for participants to recognize even synthesized micro-expressions (e.g., Shen et al., 2012, 2016a,b; Hurley et al., 2014; Svetieva and Frank, 2016). Thus, it would be very helpful for researchers and practitioners if the accuracy of micro-expression perception could be improved by training.

Scientists have long endeavored to train people to better recognize these facial expressions. The Japanese and Caucasian Brief Affect Recognition Test (JACBART) was the first rigorously validated test of micro-expression recognition accuracy (Matsumoto et al., 2000). This test was further evolved into a self-instructional training package, called the Micro Expression Training Tool (METT; Ekman, 2002; Matsumoto and Hwang, 2011; Hurley, 2012; Hurley et al., 2014). It has been used to improve the recognition accuracy of university students, school teachers, business persons, Coast Guard personnel, and even patients diagnosed with schizophrenia (Russell et al., 2006, 2008; Endres and Laidlaw, 2009; Frank et al., 2009a; Marsh et al., 2010; Matsumoto and Hwang, 2011). Studies even found that, after the METT training, the communicative skills of participants had also been improved, and this training effect could be retained for 2–3 weeks (Matsumoto and Hwang, 2011). However, evidence also indicates that, in the current situation, the validity of micro-expression training programs is very limited when putting it into practice. Results showed that, even after the METT training, the accuracy for recognizing real-world spontaneous micro-expressions was still less than 40%, which renders this tool inefficient in practice (Frank et al., 2009a).

To develop more efficient training tools, it is the prime necessity for researchers to have a more complete knowledge about the process of micro-expression recognition. Little is known regarding this process. Only a few studies have investigated this subject. For example, researchers found that, factors like emotional context, duration of facial expressions, age, personality, or profession can affect micro-expression recognition (e.g., Matsumoto et al., 2000; Hall and Matsumoto, 2004; Hurley, 2012; Shen et al., 2012; Hurley et al., 2014; Zhang et al., 2014, 2018; Shen et al., 2016b; Svetieva and Frank, 2016; Demetrioff et al., 2017). Evidence also suggests that there are different EEG/ERPs neural mechanisms underlying the recognition of micro-expressions and macro-expressions (i.e., the typical facial expressions, usually last between 0.5 and 4 s) (Shen et al., 2016a). The brain regions responsible for the differences might be the inferior temporal gyrus and the frontal lobe. Furthermore, the left hemisphere was more involved in processing micro-expressions (Shen et al., 2016a).

Besides the typical visual routes to emotion recognition, researchers found that people might also make use of sensorimotor simulation, in which the motor production of the perceived facial expression was recreated to facilitate emotion perception (e.g., Oberman et al., 2007; Niedenthal et al., 2010; Hawk et al., 2012; Price and Harmon-Jones, 2015; for review, see Wood et al., 2016a). Growing evidence indicates that people automatically and regularly mimic facial expressions (e.g., Tamietto et al., 2009; Neal and Chartrand, 2011; Bornemann et al., 2012; Korb et al., 2014). These subtle muscle contractions in the perceiver’s face generate afferent facial feedback signals to the brain and then the perceiver uses these feedback signals to reproduce and thus recognize others’ facial expressions (Wood et al., 2016a). Recent studies show that facial feedback can affect macro-expression recognition. When facial feedback is temporarily blocked (by chewing, biting, or intentionally preventing mimicry), the perceiver’s accuracy of macro-expression recognition is usually lower than normal (e.g., Oberman et al., 2007; Stel and van Knippenberg, 2008; Neal and Chartrand, 2011; Ponari et al., 2012; Hyniewska and Sato, 2015; Ipser and Cook, 2015; for review, see Wood et al., 2016a). Study also showed that amplifying facial feedback through a gel facemask improved the recognition performance for difficult macro-expression recognition tasks, such the Reading the Mind in the Eyes Test (Neal and Chartrand, 2011). These studies demonstrate that the facial feedback produced by facial muscles that occurs involuntarily during perception of one’s facial expression may later contribute to the efficient and accurate recognition of that facial expression.

Can facial feedback also contribute to the recognition of micro-expression? Understanding this question would help the researchers to extend the embodied theory of emotion perception, to design better micro-expression recognition tools, and to develop more efficient deception detection methods. Recent studies have already shed some light on this question and they suggest that the answer might be “yes.” Researchers found that the facial mimicry occurs even in response to expressions of which the perceiver is unaware (e.g., Dimberg et al., 2000; Tamietto et al., 2009; Hess and Fischer, 2014), indicating that the facial feedback signals also exist during the perception of micro-expressions. One recent study directly found the evidence that facial feedback signals do participate in the recognition of micro-expressions. Similar to the study of Neal and Chartrand (2011), by applying a restricting gel composed of polyvinyl alcohol and polyvinylpyrrolidone to the full face, the researchers amplified the facial feedback signals during the micro-expression recognition process (Wu et al., 2016). Results showed that, the recognition accuracy of micro-expressions with high intensity in facial actions (termed as intense micro-expressions in that study) was unaffected by the gel applying procedure, but the recognition accuracy was impaired for micro-expressions that were low in the intensity level of facial actions (termed as subtle micro-expressions in that study) after wearing the gel. This study indicates that actually facial feedback is a deleterious cue for micro-expression recognition. However, given that this study utilize the full face as the target area, it is difficult for the researchers to identify the main source region of that deleterious effect.

Studies showed that there were different perceptual weights between different face areas during emotion perception process. The superiority of the lower face area had been found in the perception of macro-expressions (e.g., Calder et al., 2000; Cui et al., 2009; Calvo et al., 2013a; Mu et al., 2017). For example, the picture of a smiling mouth was found to bias perceived expression in upper face toward happiness, even when the actual upper face was not happy (Kontsevich and Tyler, 2004; Calvo et al., 2013a,b). By employing electromyography (EMG), one recent study on facial feedback also provides the indirect evidence to suggest that, during explicit facial imitation of macro-expressions, facial muscles on the lower face (e.g., depressor) might be more activated than the upper facial muscles (e.g., corrugator, frontalis; Wingenbach et al., 2018). Additional evidence suggests that the lower face also has more weight in the perception of micro-expressions. Studies have found that micro-expressions on the lower face inhibited conscious detection of all types of micro-expressions on the upper face, even when viewers had paid full attention to the upper face (Iwasaki and Noguchi, 2016). These results are interesting because these results showed that the lower face seems to play a more important role in facial expression recognition. Consequently, combined with the studies on facial feedback, these results suggest that during the micro-expression recognition process, facial feedback from the lower face is the main source of disruption. That is, facial feedback signals from different partial face areas may have different effects on micro-expression recognition: While facial feedback from the lower face is deleterious for micro-expression recognition, facial feedback from the upper face may still be beneficial. Therefore, amplifying the facial feedback from the upper face may boost the recognition accuracy for micro-expressions, but amplifying the facial feedback from the lower face may impair people’s ability to read micro-expressions. More importantly, temporarily blocking the facial feedback from the lower face should be able to improve the micro-expression recognition accuracy for participants.

In the current study, we investigated the effects of partial facial feedback on micro-expression recognition by three behavioral experiments. Specifically, in Study 1 and 2, we tested the effects of amplified partial facial feedback on the recognition of intense micro-expressions (Study 1) and subtle micro-expressions (Study 2)^1^. In Study 3, we tested the effects of blocking facial feedback of lower face on the recognition of both intense and subtle micro-expressions. Consistent with previous studies, the division of the face into upper and lower facial regions was accomplished by holding a rectangular piece of cardboard in front of the area to be located. The horizontal edge of the upper face was located slightly below the bridge of the nose (e.g., Bassili, 1979; Calvo et al., 2013a,b). In addition, the upper face ranged from this horizontal edge to the hairline, and the lower face ranged from this horizontal edge to the chin.

## Study 1

Given that the amplitude of the facial actions of intense micro-expressions is high, decoding the movement of intense micro-expressions should be relatively easier. In Study 1, we investigated the effects of amplifying partial facial feedback signals (from upper face or lower face) on the recognition of intense micro-expressions.

The previous study (Wu et al., 2016) has shown that facial feedback signals of the full face were ineffective cues for the recognition of intense micro-expressions. It should be noted that the duration upper limit in that study (Wu et al., 2016) was only about 1/3 s. However, researchers have reported that, for a typical micro-expression, its duration could be much longer (about 1/2 s; Matsumoto and Hwang, 2011; Yan et al., 2013). Hence, in Study 1, we employed four different settings of duration (50, 150, 333, and 450 ms) to test our hypothesis.

### Method

#### Participants and Design

G^∗^Power Version 3.1.9.2 software (Faul et al., 2009) was used to acquire an a priori estimate of the required sample size. Using the parameters (power = 0.8, effect size *f* = 0.25, α = 0.05)^2^ and giving the current experimental design, the analysis estimated a sample size of 102. We finally recruited a total of 132 undergraduates (*M*_age_ = 22.7, *SD* = 1.79, 37 males and 78 females). The actual power for this sample size is 0.9. This study was carried out in accordance with the recommendations of the IRB of the Institute of Psychology, Hunan Normal University, with written informed consent from all participants. All participants gave written informed consent in accordance with the Declaration of Helsinki. The protocol was approved by the IRB of the Institute of Psychology, Hunan Normal University.

A 3 (partial facial feedback: upper face, lower face, control) × 4 (duration: 50, 150, 333, and 450 ms) mixed-model experimental design was used, with partial facial feedback being the between-subjects factor and duration being the within-subjects factor.

#### Facial Feedback Manipulation

We employed the method of Neal and Chartrand (2011) to manipulate facial feedback. This manipulation relies on a well-established principle of proprioceptive feedback whereby afferent muscle signals are amplified when the initiating muscle meets resistance or load during contraction (e.g., Gandevia and Burke, 1992; Allen et al., 2008). Similar to previous studies, in order to create resistance in response to facial muscle contractions, we used a gel composed of polyvinyl alcohol and polyvinylpyrrolidone, which dry and contract upon exposure to air in approximately 10 min (Neal and Chartrand, 2011; Wood et al., 2016b). This restricting gel manipulation can amplify facial feedback signals by preserving the initiation of muscle movements but increasing participants’ subjective experience of resistance to these movements. Participants were randomly assigned to one of the three partial facial feedback conditions, and all participants were asked to apply the restricting gel to their faces or their non-dominant inner arms in a thick layer.

**FIGURE 1:**
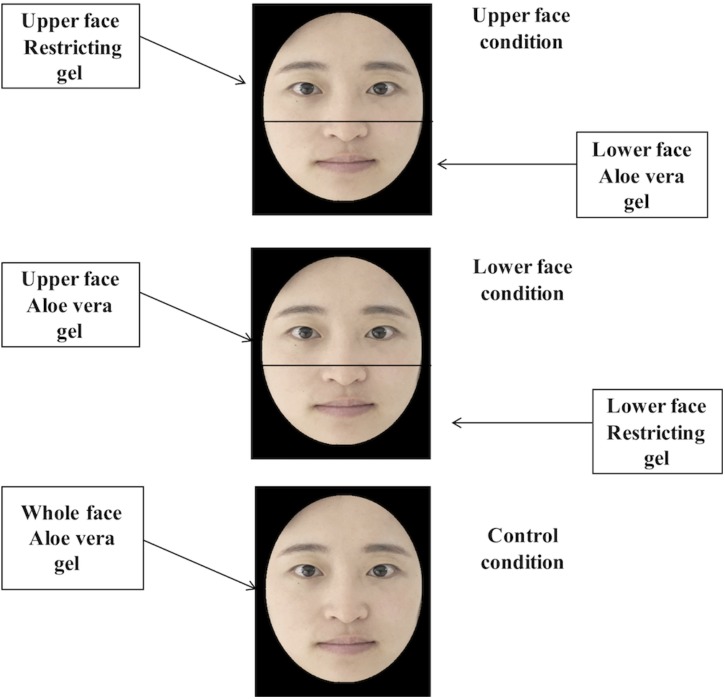
The division of upper and lower faces and the gel applying procedures on the face. Note that we use the facial images of the first author for illustration.

Specifically, as for participants who were randomly assigned to the upper face condition, they were asked to apply the restricting gel to their upper faces, while a moisturizer (aloe vera gel) was applied to their lower faces and the restricting gel was also applied to their non-dominant inner arms. Contrary to the upper face condition, when participants were randomly assigned to the lower face condition, they were asked to apply the restricting gel to their lower faces while a moisturizer (aloe vera gel) was applied to their upper faces. Similarly, the restricting gel was also applied to their non-dominant inner arms. The division of the face into upper and lower facial regions was accomplished by holding a rectangular piece of cardboard in front of the area to be masked. The horizontal edge of the mask was placed slightly below the bridge of the nose (e.g., Bassili, 1979; Calvo et al., 2013a,b). In addition, the upper face ranged from this horizontal edge to the hairline, and the lower face ranged from this horizontal edge to the chin. As for participants who were randomly assigned to the control condition, a moisturizer (aloe vera gel) was applied to their whole faces and the restricting gel was applied to their non-dominant inner arms. These manipulations were simultaneously performed by three independent research assistants (one for the upper face, one for the lower face, and one for the arm). The three assistants finished their procedures in approximate time, and participants had their eyes closed during the gel applying procedures (see Figure 1).

#### Micro-Expression Recognition Task and Working Memory Task

**FIGURE 2:**
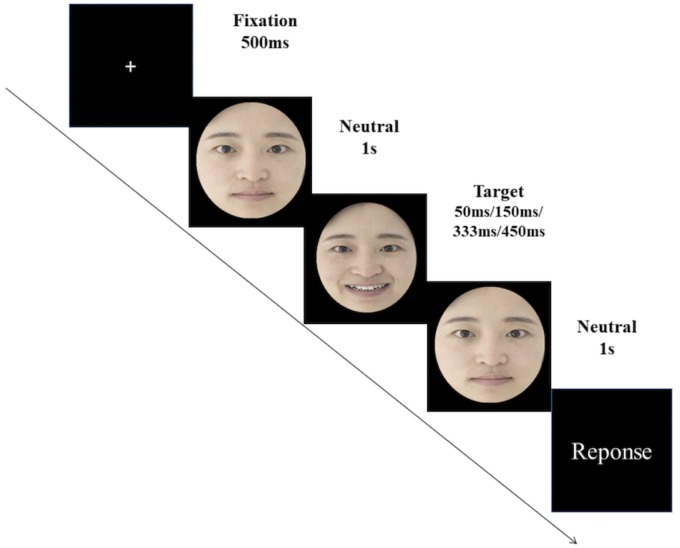
The JACBART paradigm. Note that we use the facial images of the first author for illustration.

Twelve models from the BU-3DFE database were randomly selected (Yin et al., 2006). The database contains 100 models (56 female, 44 male), with ages ranging from 18 to 70 years old, and a variety of ethnic origins including White, Black, East-Asian, Middle-East Asian, Indian and Hispanic. Each model posed seven facial expressions, which include neutral and six universal facial expressions (sadness, surprise, anger, disgust, fear, and happiness) with four different intensity levels in generalized facial actions (low, middle, and high, very high). For each of these selected models, the images of his/her six basic facial expressions (i.e., sadness, surprise, anger, disgust, fear, and happiness) and the image of his/her neutral face were selected as the stimulus materials for Study 1. Therefore, a total of 84 facial images were selected, and we only selected the facial expressions (excluding the neutral faces) that were rated to be “very high” in the intensity level of facial expressions (Yin et al., 2006). The selected models were randomly divided into four model sets, therefore in each set there were three models. Then these four model sets were randomly assigned to the four duration conditions. All of the six categories of micro-expressions of each model (happiness, disgust, fear, angry, surprise, and sadness) were presented according to his/her assigned duration condition (i.e., presented for 50, 150, 333, or 450 ms). The order of the combination of the model sets and the duration conditions was counterbalanced across participants by balanced Latin square. The micro-expressions were presented by employing the JACBART paradigm (Matsumoto et al., 2000; Matsumoto and Hwang, 2011; Shen et al., 2012; Zhang et al., 2014, 2018; Svetieva and Frank, 2016), in which the expression image (presented for 50, 150, 333, or 450 ms) was sandwiched in between two 1 s presentations of the same expresser’s neutral face (see Figure 2). After that, participants were asked to select a single emotion label from a list provided that corresponded to the expression just displayed. The response options included sadness, surprise, anger, disgust, fear, happiness, and an option of “none of the above.” The presentation order of the stimulus was completely randomized, and each micro-expression of each model was presented only once. Therefore there were 72 trials in total. The accuracy data were recorded for this task.

Since it was possible that some of the facial feedback manipulation procedures were more distracting or consumed more cognitive resources than the others, and thus confounding with the effects of facial feedback, participants were also asked to finish a working memory task, in which participants had to finish 16 modular arithmetic (MA) questions that had been shown to be highly sensitive to variations in cognitive loads (Beilock et al., 2004). The data of accuracy and reaction time (RT) were recorded. This working memory task was directly adopted from the study of Neal and Chartrand (2011).

#### Procedure

Participants were told to take part in two unrelated studies. They were told that the first study was a trial study of a new cosmetic product. Upon arrival at the event site, participants were asked to finish the an questionnaire, in which we asked the participants to answer the following questions: (a) whether now they could contract their upper face/lower face/non-dominant inner arm muscles (yes/no) and (b) whether now their skin of upper face/lower face/non-dominant inner arm felt resistant to underlying muscle movements (11-point scale, from “not resistant at all” to “extremely resistant”) and (c) the humidity they felt on their skin of corresponding areas (upper face/lower face/inner arm) (10-point scale, from “very dry” to “very wet”). After completing this questionnaire, participants were randomly assigned to one of the three partial facial feedback conditions, then the partial feedback manipulations were delivered. After wearing the gel for 10 min, participants were required to fill another questionnaire, in which the contents were almost identical to those of the first questionnaire except that an extra question about the comfortableness they felt on their skin of corresponding areas (upper face/lower face/inner arm) after applying the gel (10-point scale, from “extremely uncomfortable” to “extremely comfortable”) was included. Then, participants were told that they had to wait for a while before being asked about the effects of the cosmetics, and in the meanwhile, they had to complete another study which was an emotion task that had nothing to do with the cosmetics. Then participants were asked to finish the micro-expression recognition task and the working memory task.

### Results and Discussion

All participants reported being able to move the relevant muscles before or after applying the gel. The rating scores of the resistance were subjected to a 2 (gel applying time: before, after) × 3 (partial facial feedback: upper face, lower face, control) × 3 (region: upper face, lower face, non-dominant inner arm) mixed-model analysis of variance (ANOVA). The results showed that the main effect of partial facial feedback [*F*(2,129) = 72.46, *p* < 0.001, $\eta_{p}^{2}$ = 0.53], and the main effect of gel applying time [*F*(1,129) = 3684.2, *p* < 0.001, $\eta_{p}^{2}$ = 0.97] were significant, meanwhile the interaction between partial facial feedback and gel applying time were significant [*F*(2,129) = 145.89, *p* < 0.001, $\eta_{p}^{2}$ = 0.69]. Moreover, the main effect of region were significant [*F*(2,258) = 533.94, *p* < 0.001, $\eta_{p}^{2}$ = 0.81], and the interactions of all these three independent variables were significant [*F*(4,258) = 582.74, *p* < 0.001, $\eta_{p}^{2}$ = 0.9]. The interaction between region and gel applying time were significant [*F*(2,258) = 920.44, *p* < 0.001, $\eta_{p}^{2}$ = 0.88] and the interaction between the partial facial feedback and region were significant [*F*(4,258) = 318.54, *p* < 0.001, $\eta_{p}^{2}$ = 0.83].

Further simple effects analysis showed that ratings of resistance of the target regions (i.e., the upper face and arm in upper face group, the lower face and arm in lower face group, and the arm in control group) were significantly increased after wearing the gel (*F*s > 1179.63, *p*s < 0.001; see Table 1), and the facial feedback manipulation only led to increased ratings of resistance in the target regions (*F*s < 0.18, *p*s > 0.67). In addition, after wearing the gel mask, the rating of resistance of upper face in the upper face group was significantly higher [*F*(2,129) = 756.44, *p* < 0.001, $\eta_{p}^{2}$ = 0.92] than those in the lower face (*p* < 0.001) and control (*p* < 0.001) groups, and the rating of resistance of lower face in the lower face group was significantly higher [*F*(2,129) = 838.2, *p* < 0.001, $\eta_{p}^{2}$ = 0.93] than those in the upper face (*p* < 0.001) and control (*p* < 0.001) groups. The differences of resistance in the arm among three partial facial feedback conditions before or after wearing the gel were not significant (*F*s < 0.17, *p*s > 0.85). There were no significant differences in the ratings of resistance among the non-target facial regions after wearing the gel (within each partial facial feedback condition or among the three partial facial feedback conditions). The baselines before wearing the gel and the rating of resistance after wearing the gel in each target facial region of each partial facial feedback condition were also homogeneous (*p*s > 0.72; for more detailed statistics, see Supplementary Material). These results indicated that the partial facial feedback manipulations were successful. They increased the facial feedback from the upper face and the lower face without incurring other confounds.

**Table 1:** The rating of resistance before and after facial feedback manipulation (Study 1).

	Upper face group				Lower face group				Control			
	Before		After		Before		After		Before		After	
	*M*	*SD*	*M*	*SD*	*M*	*SD*	*M*	*SD*	*M*	*SD*	*M*	*SD*
Upper face	2.02	0.7	7.32	0.74	2.09	0.71	2.11	0.72	2	0.75	2.02	0.73
Lower face	2.07	0.7	2.02	0.7	2	0.75	7.34	0.71	2.07	0.7	2.02	0.7
Arm	2.05	0.71	7.34	0.71	2.09	0.74	7.36	0.72	2	0.75	7.34	0.71


**FIGURE 3:**
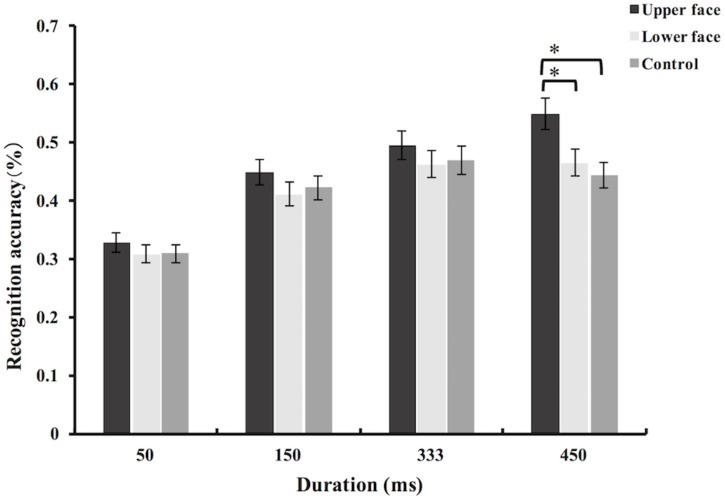
Mean recognition accuracies of intense micro-expressions in Study 1. Error bars represent standard errors. The symbol ^∗^ indicates that the differences were significant.

The accuracy data of micro-expression recognition task were then analyzed using mixed-model ANOVA. The main effect of partial facial feedback [*F*(2,129) = 3.75, *p* = 0.03, $\eta_{p}^{2}$ = 0.06], the main effect of duration [*F*(3,387) = 96.52, *p* < 0.001, $\eta_{p}^{2}$ = 0.43] and the interaction between partial facial feedback and duration [*F*(6,387) = 2.22, *p* = 0.04, $\eta_{p}^{2}$ = 0.03] were all significant. Simple effects analysis revealed that the effect of partial facial feedback was significant in the condition of 450 ms [*F*(2,129) = 11.45, *p* < 0.001, $\eta_{p}^{2}$ = 0.15]. However, there were no significant differences on the recognition accuracy among the different partial facial feedback groups under the durations of 50, 150, and 333 ms [*F*s < 1.14, *p*s > 0.32] (see Figure 3). Further *post hoc* comparisons (Bonferroni) showed that, under the condition of 450 ms, the recognition accuracy of the upper face group was significantly increased compared with the lower face group [*t*(129) = 3.39, *p* = 0.002] and control group [*t*(129) = 4.52, *p* < 0.001]. But, the difference between lower face group and control group was not significant under the condition of 450 ms [*t*(129) = 1.09, *p* = 0.8]. To explore the potential moderation of category of micro-expression on the effects of partial facial feedback, we further added the factor of emotion category (i.e., sadness, surprise, anger, disgust, fear, and happiness) into analysis. The results showed that the interaction between emotion category and partial facial feedback, and the interaction of emotion category, partial facial feedback, and duration, were all not significant (*F*s < 1.17, *p*s > 0.26).

The accuracy and RT data of the working memory task were analyzed using one-way ANOVA. Results showed that, there were no significant differences in accuracy [*F*(2,129) = 1.62, *p* = 0.2, $\eta_{p}^{2}$ = 0.03] or in RTs [*F*(2,129) = 0.3, *p* = 0.74, $\eta_{p}^{2}$ = 0.005] among the upper face, lower face and control conditions (see Table 2). Thus, the results found in the micro-expression recognition task of Study 1 cannot be attributed to the differences in cognitive load caused by facial feedback manipulations.

**Table 2:** The reaction time and accuracy of working memory task (Study 1).

	Upper face group		Lower face group		Control	
	*M*	*SD*	*M*	*SD*	*M*	*SD*
Accuracy	0.66	0.20	0.65	0.20	0.72	0.15
RT(ms)	6402	1903	6388	1675	6641	1552


In summary, the results of Study 1 showed that participants had better judgment of intense micro-expressions with a duration of 450 ms when their skin of upper face had been made resistant to underlying muscle contractions via a restricting gel. Previous studies on embodied cognition found that facial feedback might aid emotion perception (e.g., Ipser and Cook, 2015; Lobmaier and Fischer, 2015; Kaiser and Gcl, 2017). Researchers found the existence and influence of facial feedback upon numerous cognitive processes, such as recognition of subtle or ambiguous expressions, evaluation of the emotional meanings of expressions, language comprehension, and etc. (Davis et al., 2010; Havas et al., 2010; Rychlowska et al., 2014). Similar to these studies, the results of Study 1 also discovered the participation of facial feedback during the perception of one’s actual social intentions (i.e., the interpretation of one’s intense micro-expressions). They were consistent with our prediction and thus provide partial support to our hypothesis.

## Study 2

In study 1, we investigated the role of partial facial feedback played during the recognition of intense micro-expressions. However, researchers have found that typical micro-expressions are usually low in intensity (Porter and ten Brinke, 2008; Yan et al., 2013). Therefore, in Study 2, we further investigated the effects of amplifying partial facial feedback signals (from upper face or lower face) on the recognition of subtle micro-expressions. The recent evidence showed that facial feedback signals from the full face are disruptive cues for the recognition of subtle micro-expressions (Wu et al., 2016). Growing evidence also found that the lower face has more weight in facial expression recognition (e.g., Calvo et al., 2013a; Iwasaki and Noguchi, 2016), which suggest that the lower face is the main source of disruption for the recognition of subtle micro-expressions. Therefore, we predicted that amplifying facial feedback of the lower face can reduce the recognition accuracy for subtle micro-expressions.

### Method

#### Participants and Design

G^∗^Power Version 3.1.9.2 software (Faul et al., 2009) was used to acquire an a priori estimate of the required sample size. Consistent with Study 1, we also used *f* = 0.25 as our effect size estimation. Using the parameters (power = 0.8, effect size *f* = 0.25, α = 0.05) and giving the current experimental design, the analysis estimated a sample size of 102. We finally recruited a total of 108 undergraduates (*M*_age_ = 18.69, *SD* = 1.58, 37 males and 71 females). This study was carried out in accordance with the recommendations of the IRB of the Institute of Psychology, Hunan Normal University, with written informed consent from all participants. All participants gave written informed consent in accordance with the Declaration of Helsinki. The protocol was approved by the IRB of the Institute of Psychology, Hunan Normal University.

A 3 (partial facial feedback: upper face, lower face, control) × 4 (duration: 50, 150, 333, and 450 ms) mixed-model experimental design was used, with partial facial feedback being the between-subjects factor and duration being the within-subjects factor.

#### Materials and Procedure

In Study 2, the manipulation of the facial feedback, the experiment tasks and the procedure were exactly the same as those of Study 1, except that the facial stimulus of Study 2 was very low in the intensity level. These facial expressions were selected from twelve models of BU-3DFE database as in Study 1, and we only selected the facial expressions (excluding the neutral faces) that were rated to be “low” in the intensity level from this database (Yin et al., 2006).

### Results and Discussion

All participants reported being able to move the relevant muscles at both time points (before or after applying the gel). The rating scores of the resistance were subjected to a 3 (gel applying time: before, after) × 3 (partial facial feedback: upper face, lower face, control) × 3 (region: upper face, lower face, the non-dominant inner arm) mixed-model ANOVA. The results showed that, the main effect of partial facial feedback [*F*(2,105) = 56.51, *p* < 0.001, $\eta_{p}^{2}$ = 0.52], and the main effect of gel applying time [*F*(1,105) = 3399.6, *p* < 0.001, $\eta_{p}^{2}$ = 0.97], were significant. The interaction between partial facial feedback and gel applying time was also significant [*F*(2,105) = 142.34, *p* < 0.001, $\eta_{p}^{2}$ = 0.73]. In addition, the main effect of region was significant [*F*(2,210) = 540.58, *p* < 0.001, $\eta_{p}^{2}$ = 0.84], the interaction between region and gel applying time [*F*(2,210) = 689.82, *p* < 0.001, $\eta_{p}^{2}$ = 0.87], and the interaction between partial facial feedback and region were significant [*F*(4,210) = 347.35, *p* < 0.001, $\eta_{p}^{2}$ = 0.87]. More importantly, we also observed a significant interaction of these three independent variables [*F*(4,210) = 452.36, *p* < 0.001, $\eta_{p}^{2}$ = 0.9] (see Table 3).

**Table 3:** The rating of resistance before and after facial feedback manipulation (Study 2).

	Upper face group				Lower face group				Control			
	Before		After		Before		After		Before		After	
	*M*	*SD*	*M*	*SD*	*M*	*SD*	*M*	*SD*	*M*	*SD*	*M*	*SD*
Upper face	2.00	0.68	7.44	0.74	1.94	0.72	2.00	0.72	1.97	0.69	2.03	0.7
Lower face	2.08	0.65	2.14	0.64	1.97	0.7	7.39	0.69	2.03	0.7	2.11	0.67
Arm	2.03	0.65	7.39	0.73	1.92	0.69	7.33	0.72	2.11	0.67	7.31	0.71


Further simple effects analysis showed that, consistent with Study 1, ratings of resistance of the target regions (i.e., the upper face and arm in upper face group, the lower face and arm in lower face group, and the arm in control group) were significantly increased after wearing the gel (*F*s > 1018.26, *p*s < 0.001), and the facial feedback manipulation only led to increased ratings of resistance in the target regions (*F*s < 0.25, *p*s > 0.62). In addition, after wearing the gel mask, the rating of resistance of upper face in the upper face group was significantly higher [*F*(2,129) = 689.91, *p* < 0.001, $\eta_{p}^{2}$ = 0.93] than those in the lower face (*p* < 0.001) and control (*p* < 0.001) groups, and the rating of resistance of lower face in the lower face group was significantly higher [*F*(2,129) = 752.18, *p* < 0.001, $\eta_{p}^{2}$ = 0.94] than those in the upper face (*p* < 0.001) and control (*p* < 0.001) groups. The differences of resistance in the arm among three partial facial feedback conditions before or after wearing the gel were not significant (*F*s < 0.76, *p*s > 0.47). More importantly, there were no significant differences in the ratings of resistance among the non-target facial regions before or after wearing the gel (within each partial facial feedback condition or among the three partial facial feedback conditions), and the baselines and the ratings of resistance after wearing the gel in each target facial region of each partial facial feedback condition were also homogeneous (*p*s > 0.99; for detailed statistics, see Supplementary Material). Therefore, the results showed that consistent with Study 1, the partial facial feedback manipulations of Study 2 were also successful.

**FIGURE 4:**
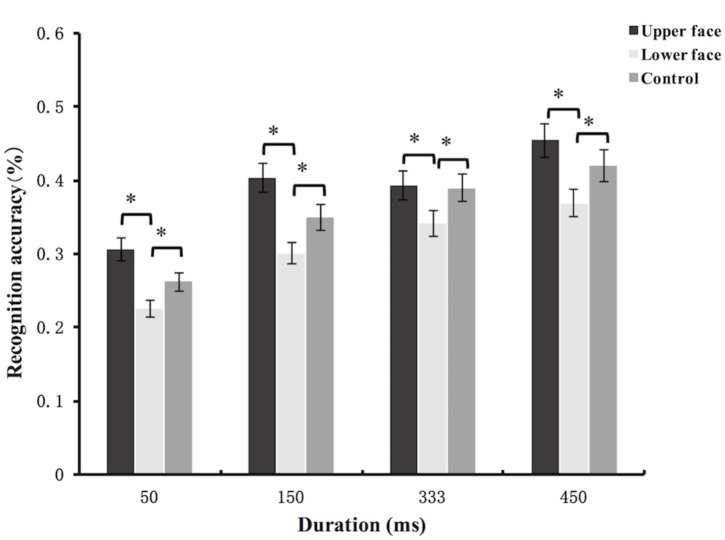
Mean recognition accuracies of subtle micro-expressions in Study 2. Error bars represent standard errors. The symbol ^∗^ indicates that the differences were significant.

The accuracy data of micro-expression recognition task were analyzed using mixed-model ANOVA. The main effect of partial facial feedback [*F*(2,105) = 11.33, *p* < 0.001, $\eta_{p}^{2}$ = 0.18], and the main effect of duration were significant [*F*(3,315) = 63.23, *p* < 0.001, $\eta_{p}^{2}$ = 0.38]. The interaction between partial facial feedback and duration [*F*(6,315) = 0.86, *p* = 0.52, $\eta_{p}^{2}$ = 0.02] were not significant. Further *post hoc* comparisons (Bonferroni) showed that compared with the upper face group [*t*(105) = -4.71, *p* < 0.001] and the control group [*t*(105) = -2.71, *p* = 0.02], the recognition accuracy of lower face group was significantly decreased. In addition, the difference of recognition accuracy between upper face group and control group was not significant [*t*(105) = 2, *p* = 0.14] (see Figure 4). To explore the potential moderation of category of micro-expression on the effects of partial facial feedback, we further added the factor of emotion category into analysis. The results showed that the interaction between emotion category and partial facial feedback, and the interaction of emotion category, partial facial feedback, and duration, were all not significant (*F*s < 1, *p*s > 0.48).

Consistent with Study 1, the partial facial feedback manipulations in Study 2 brought similar change in cognitive load (see Table 4). The results of the working memory task showed that there was no significant main effect of partial facial feedback manipulation on accuracy [*F*(2,105) = 0.12, *p* = 0.89, $\eta_{p}^{2}$ = 0.002] or on RT [*F*(2,105) = 0.2, *p* = 0.82, $\eta_{p}^{2}$ = 0.004]

**Table 4:** The reaction time and accuracy of working memory task (Study 2).

	Upper face group		Lower face group		Control	
	*M*	*SD*	*M*	*SD*	*M*	*SD*
Accuracy	0.63	0.16	0.64	0.16	0.65	0.19
RT(ms)	6030	1684	6299	1836	6132	2009


In sum, the results of Study 2 showed that amplifying facial feedback of lower face decreased the recognition accuracy of subtle micro-expressions. These results were consistent with our prediction, they indicated that facial feedback from the lower face was a deleterious cue for the recognition of subtle micro-expressions.

## Study 3

According to our hypothesis, facial feedback from the lower face is the deleterious clue for the recognition of micro expressions. In Study 1 and Study 2, we tested the hypothesis by amplifying the facial feedback signals. If our hypothesis is correct, blocking facial feedback of lower face would increase the recognition accuracy of micro-expressions. This possibility was tested in Study 3.

According to the results of Studies 1 and 2, the effects of amplifying the facial feedback from the lower face did not differ when the duration of micro-expressions was changed (i.e., 50, 150, 333, and 450 ms). Therefore, we set the presentation time of micro-expressions to be 150 and 333 ms in Study 3.

### Method

#### Participants and Design

G^∗^Power Version 3.1.9.2 software (Faul et al., 2009) was used to acquire an a priori estimate of the required sample size. Effect size was estimated according to the study of Rychlowska et al. (2014). We employ their minimum effect size (*f* = 0.33; obtained by blocking the facial mimicry through mouthguard as in this Study 3) as our estimation (see Rychlowska et al., 2014). The power analysis (power = 0.8, α = 0.05) estimated a sample size of 78. We finally recruited a total of 80 undergraduates (*M*_age_ = 19.2, *SD* = 1.67, 12 males and 68 females). This study was carried out in accordance with the recommendations of the IRB of the Institute of Psychology, Hunan Normal University, with written informed consent from all participants. All participants gave written informed consent in accordance with the Declaration of Helsinki. The protocol was approved by the IRB of the Institute of Psychology, Hunan Normal University.

A 2 (facial feedback: lower face blocked, control) × 2 (duration: 150 ms, 333 ms) × 2 (intensity: subtle, intense) mixed-model experimental design was used, with facial feedback being the between-subjects factor while duration and intensity being the within-subjects factors.

#### Facial Feedback Manipulation

**FIGURE 5:**
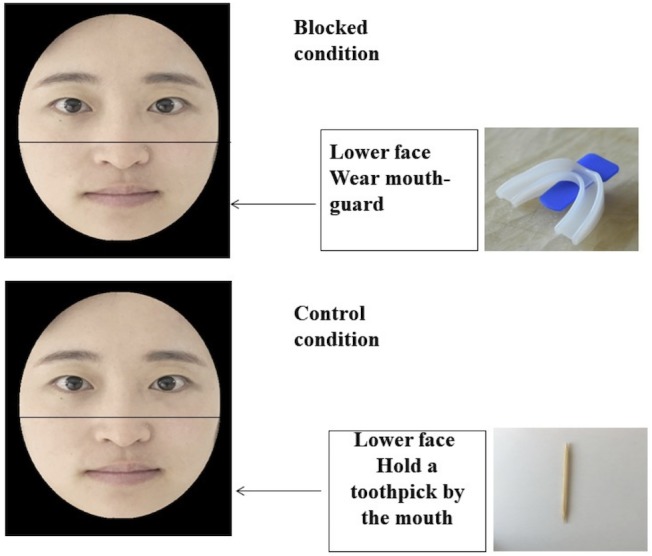
Facial feedback manipulation procedures in Study 3. Note that we use the facial images of the first author for illustration.

Participants were randomly assigned to one of the two facial feedback conditions. Specifically, as for participants who were randomly assigned to the lower face blocked condition, each participant received a new, transparent “boil and bite” mouthguard (the top-bottom type), sealed in an unopened box. We provided the participants with hot and cold water, along with the instructions on how to properly wear the mouthguard using tongue and biting pressure. All participants were asked to follow the instructed procedure and were asked to wear the mouthguard during the whole experiment (see Figure 5). By recording the EMG activity of participants’ facial muscles, previous study has demonstrated that this manipulation is a valid procedure for blocking facial feedback of lower face (Rychlowska et al., 2014). When participants were randomly assigned to the control condition, they were asked to finish the experiment with a toothpick in the mouth and hold it by putting the tip of toothpick in the interdental space and closing the mouth.

#### Micro-Expression Recognition Task and Working Memory Task

Twelve models from the BU-3DFE database were randomly selected (Yin et al., 2006). For each model, the images of his/her six basic facial expressions and the image of his/her neutral face were selected as the stimulus materials for Study 3. Facial expressions of two intensity levels (“very high” and “low”) were selected for each model (Yin et al., 2006) and a total of 168 facial images were selected. The selected models were randomly divided into four model sets, therefore in each set there were three models. Then these four model sets were randomly assigned to the four experimental conditions. For each model, his/her six micro-expressions were presented according to his/her assigned experimental condition (e.g., intense/150 ms). The order of the combination of the model sets and the experimental conditions was counterbalanced across participants by balanced Latin square. The micro-expressions were presented by employing the JACBART paradigm as in Study 1. The presentation order of the stimulus was completely randomized, and micro-expression of each model was presented only once. Therefore there were 72 trials in the micro-expression recognition task. The working memory task was exactly the same as that of Study 1.

#### Procedure

Participants were tested individually via the computer by using the Eprime 2.0 software. They were randomly assigned to one of the two facial feedback conditions. Specifically, as for participants who were randomly assigned to the lower face blocked condition, they were asked to wear mouthguards to finish the experiment. Participants in the control condition had to hold a toothpick by their teeth during the whole experiment, and all participants were told to keep the state until the end of the experiment. Then participants were asked to finish the micro-expression recognition task and the working memory task.

### Results and Discussion

**FIGURE 6:**
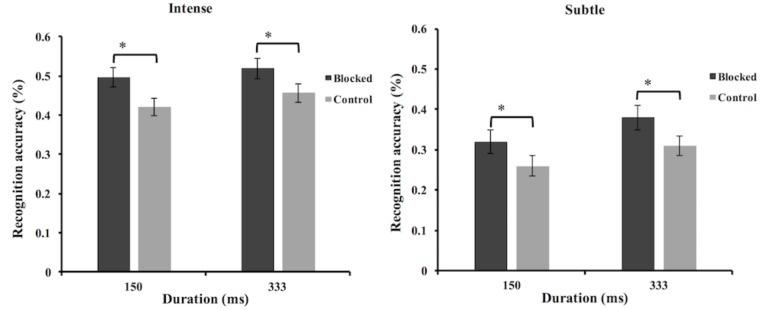
Mean recognition accuracies of subtle and intense micro-expressions in Study 3. Error bars represent standard errors. The symbol ^∗^ indicates that the differences were significant.

The micro-expression recognition accuracy data were analyzed using mixed-model ANOVA. The results showed that the main effect of facial feedback [*F*(1,78) = 13.09, *p* = 0.001, $\eta_{p}^{2}$ = 0.14], and the main effect of duration [*F*(1,78) = 8.56, *p* = 0.004, $\eta_{p}^{2}$ = 0.1] were all significant. But the interaction between facial feedback and duration [*F*(1,78) = 0.001, *p* = 0.98, $\eta_{p}^{2}$ < 0.001] was not significant. The main effect of the intensity [*F*(1,78) = 89.91, *p* < 0.001, $\eta_{p}^{2}$ = 0.54] was significant. The interaction between the facial feedback and intensity [*F*(1,78) = 0.29, *p* = 0.59, $\eta_{p}^{2}$ = 0.004], and the interaction between duration and intensity [*F*(1,78) = 1.95, *p* = 0.17, $\eta_{p}^{2}$ = 0.02] were not significant. We also found the interaction of facial feedback, intensity, and duration [*F*(1,78) = 0.29, *p* = 0.59, $\eta_{p}^{2}$ = 0.004] was not significant. In summary, the results of micro-expression recognition task showed that blocking lower face feedback increased the recognition accuracies for both intense and subtle micro-expressions. The results also showed that the recognition accuracy of intense micro-expression was higher than that of subtle micro expression, and the recognition accuracy under 333 ms condition was higher than that of 150 ms condition (see Figure 6). To explore the potential moderation of category of micro-expression on the effects of facial feedback, we further added the factor of emotion category into analysis. The results showed that the interaction between emotion category and facial feedback, the interaction of emotion category, facial feedback, and duration, and the interaction of emotion category, facial feedback, duration, and intensity, were all not significant (*F*s < 2.13, *p*s > 0.07).

The accuracy and RT data of the working memory task were analyzed using one-way ANOVA. Results showed that there were no significant differences in accuracies [*F*(1,78) = 1.05, *p* = 0.31, $\eta_{p}^{2}$ = 0.01] (lower face blocked: *M* = 0.63, *SD* = 0.16; control: *M* = 0.58, *SD* = 0.21) or in RTs [*F*(1,78) = 0.99, *p* = 0.32, $\eta_{p}^{2}$ = 0.01] (lower face blocked: *M* = 7040 ms, *SD* = 2731; control: *M* = 6462 ms, *SD* = 2454) between the two facial feedback conditions. Therefore, consistent with Study 1 and 2, the micro-expression recognition task of Study 3 cannot be attributed to the differences in cognitive load caused by facial feedback manipulations.

Consistent with our hypothesis, the results of Study 3 found that limiting facial feedback of lower face increased the recognition accuracy for both intense and subtle micro-expressions. Similar results were also found by previous studies. For example, Wood et al. (2016b) found that, amplifying the facial feedback decreased the recognition accuracy for ambiguous macro-expressions (created by morphing expressions of anger and sadness). Taken together, these studies indicate that facial feedback is not always beneficial for emotion perception, it can also be a hindrance to the recognition of expressions that are ambiguous and subtle, such as micro-expressions.

## General Discussion

By focusing on the facial displays of macro-expressions (i.e., facial expressions that last between 0.5 and 4 s; Matsumoto and Hwang, 2011), previous studies on facial feedback have consistently demonstrated that altering facial feedback impacts the emotion perception process, changing people’s ability to discriminate facial expressions (Oberman et al., 2007; Stel and van Knippenberg, 2008; Neal and Chartrand, 2011; Ponari et al., 2012; Hyniewska and Sato, 2015; Ipser and Cook, 2015; for review, see Wood et al., 2016a). However, the present work is the first to demonstrate that altering facial feedback from separate facial regions has differential effects on the recognition of micro-expressions (i.e., extremely quick facial expressions of emotion that appear on the face for no longer than 0.5 s; Frank et al., 2009b; Matsumoto and Hwang, 2011; Yan et al., 2013). In Study 1, the recognition accuracy of intense micro-expressions was significantly improved in individuals whose facial feedback of upper face had been enhanced through a restricting gel. Results showed that such an effect was modulated by the duration of micro-expressions: Only the micro-expressions that were long enough in duration (i.e., 450 ms) were able to be affected by such a procedure. No significant effects was observed across all duration conditions of intense micro-expressions when the facial feedback from the lower face was amplified.

Differential effects of partial facial feedback was also observed in Study 2. Specifically, Study 2 showed that the recognition accuracy of subtle micro-expressions was significantly impaired when facial feedback from lower face was enhanced. The results also showed that such impairing effect was consistent across all duration conditions (i.e., 50, 150, 333, and 450 ms). Furthermore, the results showed that recognition accuracy of subtle micro-expressions was unaffected when facial feedback of upper face was enhanced by the restricting gel, and this effect was also consistent across all duration conditions (i.e., 50, 150, 333, and 450 ms). It is important to note that, in Studies 1 and 2, the results showed that the skin resistance of the target facial areas was comparable between the upper face condition and the lower face condition after applying the restricting gel, which indicated that the differential effect of partial facial feedback was not due to different levels of facial feedback from the upper and the lower facial regions.

Bidirectional effects of facial feedback has been demonstrated in the studies of macro-expressions, such that the perception accuracy not only was improved when feedback was enhanced but also declined when feedback was blocked (e.g., Stel and van Knippenberg, 2008; Neal and Chartrand, 2011; for review, see Wood et al., 2016a). Similar bidirectional relationship had also been found in present study. In Study 3, we blocked the feedback signal from the lower face via a mouthguard. Results showed that participants become better judges of subtle micro-expressions under all duration conditions (i.e., 150 and 333 ms). However, surprisingly, results of Study 3 also showed that the recognition accuracy of intense micro-expressions was improved after blocking the lower face feedback. Taken together, the current results suggest that facial feedback can modulate the recognition of micro-expressions, and facial feedback from different facial regions has differential effects on micro-expression recognition. They suggest that while the facial feedback from the upper face is an effective intrinsic cue for micro-expression recognition, facial feedback from the lower face is ineffective and detrimental for this process.

Previous study showed that facial feedback cues from full face were detrimental to micro-expression recognitions only when the intensity of micro-expressions was low (Wu et al., 2016). Similar to that work, the current results also showed that the effects of facial feedback signals were modulated by the intensity of micro-expressions. Specifically, we found that facial feedback of upper face affected micro-expression recognition only when the intensity was high (but not when the intensity was low). The results also showed that dampening facial feedback of lower face could improve the recognition accuracies for both subtle and intense micro-expressions. However, amplifying these signals only impaired the recognition of subtle micro-expressions. These results suggest that the facial feedback signals from the upper face can only participate in the perception of intense micro-expressions due to the lesser magnitude of movement in the upper face. The results also suggest that the facial mimicry of other’s lower micro-expressions is sufficient enough since that it has already provided the maximal disruptive information to the recognition of micro-expressions when the intensity of expressions is high (i.e., no significant effects were observed when amplifying the facial feedback of lower face for the recognition of intense micro-expressions). To explore these possibilities, EMG recordings of the corresponding muscles (e.g., Künecke et al., 2014) of the lower and upper faces should be employed for future works.

Why are the effects of facial feedback from the upper and lower facial regions different on the recognition of micro-expressions? Although we derived our hypothesis primarily from the embodied theory of emotion perception (Wood et al., 2016a), the current study was not able to answer this question directly. However, previous studies suggest that the answer may lie in the differential brain mechanisms controlling the lower and the upper faces. Researchers found that the upper face is bilaterally controlled by the precentral gyrus and subcortical structures such as thalamus and globus pallidus, which make the movement of the upper face more spontaneous. On the other hand, muscle movement of the lower face is mainly controlled by the contralateral projections that originate in the cortical motor strip, and thus we are able to voluntarily move the muscles of lower face (e.g., Prodan et al., 2001; Wojciechowski et al., 2014; Bhushan, 2015; Frank and Svetieva, 2015). The more spontaneous movement in the upper face may lead to the more accurate mimicking by the upper face when the duration of expression is very short, since the more spontaneous mimicking in upper face may be less dependent on attention and memory (Ross et al., 2007). Consistently, previous studies found that the lower face had more perceptual weight in the perception of micro-expressions (Iwasaki and Noguchi, 2016) and the Chinese participants paid more attention to the lower part of face while interpreting facial expressions (Mu et al., 2017). In addition, the more voluntary movement in the lower face means that it relies more on attention and consciousness. Such a process may result in the inconsistent or inaccurate mimicking in the lower face when the expression is very fast, subtle, or ambiguous (Wood et al., 2016b; Wu et al., 2016; Wingenbach et al., 2018). Future studies should address these possible underlying mechanisms.

To ensure valid control of the stimuli, we employed the well-accepted paradigm of JACBART (Matsumoto et al., 2000; Ekman, 2002; Matsumoto and Hwang, 2011; Hurley, 2012; Shen et al., 2012; Hurley et al., 2014; Zhang et al., 2014, 2018; Svetieva and Frank, 2016). Although previous study suggests that the exact dynamic motion information of facial movement may not be necessary for the perception of subtle and ambiguous expressions since this information might be automatically extracted during the perception of change (Ambadar et al., 2005), the JACBART paradigm only employed 3 sequential still images which are definitely different from the facial dynamics of spontaneous micro-expressions (Yan et al., 2013). This ecological issue should be addressed in future by testing the hypothesis on more naturalistic dataset (e.g., Yan et al., 2014). Furthermore, given the relationship between reliable/versatile facial Action Units and macro-expression (Mehu et al., 2011; Mortillaro et al., 2011; Stewart et al., 2015), the interaction between facial feedback and specific facial actions (e.g., reliable Action Units like AU 6) also should be explored.

Can the effects of facial feedback be consistent for all the six categories of micro-expressions (i.e., sadness, surprise, anger, disgust, fear, and happiness)? Previous study on this subject (Wu et al., 2016) didn’t take this issue into consideration. Given that the main purpose of the current study was to investigate the effects of partial facial feedback on the overall micro-expression recognition performance, we had only employed three trials for each category of micro-expressions under each condition. By using such a design, we did not find any significant moderation effects of emotion category on facial feedback across our three studies. However, the embodied cognition accounts postulate that, during the process of emotion perception, specific facial muscle manipulation should have its specific effect on specific category of facial expressions (e.g., Oberman et al., 2007; Ponari et al., 2012; Wingenbach et al., 2018). This question should be further investigated by employing more sufficient trial numbers in the future.

Previous study showed that the facial feedback mechanism affects the visual perceptual processing rather than the semantic extraction process for macro-expressions (Wood et al., 2016b). However, in present study, the task we employed to investigate the effects of facial feedback on micro-expression processing involved the generation of emotion word labels for the face percepts. By measuring micro-expression recognition with this verbal judgment task, the current results are unable to separate the potential roles of the facial feedback for visual processing and for higher-level conceptual processing. Although the duration of micro-expressions is usually very short which makes it unlikely that facial feedback can modulate the activation of higher-order concepts within merely 500 ms (Frank et al., 2009b; Matsumoto and Hwang, 2011; Yan et al., 2013), one recent study did find that even with a duration of 40 ms, micro-expressions can be readily identified at a conceptual level (Shen et al., 2016a). On which stage does the facial feedback affect the micro-expression perception? Further research into this question is warranted.

By testing the effects of facial feedback on micro-expression, the present work contributes to the embodied theory of emotion perception by demonstrating that the effects of facial feedback are modulated by time window and intensity of expression, and by the location of facial manipulations. The present work also contributes more broadly to growing evidence that that input from the sensorimotor modality can alter the visual perception of facial expressions (Wood et al., 2016a). The present and other cross-modal interactions (Wood et al., 2016a) suggest that the human brain binds the inputs from separate modalities to produce better estimates of an external property of the stimulus.

## Conclusion

In sum, the findings of the current study develop our understanding of the effects of facial feedback on the processing of micro-expressions. They suggest that, similar to the processing of the slow and clear macro-expressions, the perception of another person’s fleeting and ambiguous micro-expressions will also trigger the same processes involved in producing the expression, but the motor production recreated in the lower face is an ineffective and detrimental clue for the recognition of micro-expressions. Our findings provide evidence in support of our hypothesis that facial feedback from the upper face is beneficial but facial feedback from the lower face is deleterious for micro-expression recognition. These findings have the potential to help the researchers develop more efficient micro-expression recognition and training tools and thus facilitate the lie detection process.

## Data Availability

The datasets generated and analyzed for this study can be found in the figshare: https://figshare.com/s/488edd8f36fe6725d1cc.

## Author Contributions

QW and XZ conceived and designed the experiments. XZ, SZ, ZL, QZ, and MZ performed the experiments. XZ, QW, SZ, ZL, QZ, and MZ analyzed the data. XZ and QW drafted the paper. All authors participated in the revising of the paper. All authors approved of the version’s publishment and agreed to be accountable for all aspects of the work.

## Conflict of Interest Statement

The authors declare that the research was conducted in the absence of any commercial or financial relationships that could be construed as a potential conflict of interest.

## Footnotes

1 Following previous study (Wu et al., 2016), for simplicity, we also called the micro-expressions that are high in the intensity of facial actions as intense micro-expressions, and called the micro-expressions that are low in intensity as subtle micro-expressions.

2 Study 1 employed the method of Neal and Chartrand (2011) to manipulate facial feedback. However, the exact procedure of Study 1 was different from their work. They only applied the restricting gel around the lower forehead, brow, and area immediately surround the eyes, and their emotion recognition task was much easier than ours. Therefore their effect size was very large (*d* = 1.31). To detect medium-large effect, in Study 1 we employed a more conservative estimation of effect size (medium effect size *f* = 0.25; Cohen, 1988).

## References

[B1] Allen T. J. Ansems G. E. Proske U. (2008). Evidence from proprioception of fusimotor coactivation during voluntary contractions in humans. *Exp. Physiol.* 93 391–398. 10.1113/expphysiol.2007.040741 18039976

[B2] Ambadar Z. Schooler J. W. Cohn J. (2005). Deciphering the enigmatic face: The importance of facial dynamics in interpreting subtle facial expressions. *Psychol. Sci.* 16 403–410. 10.1111/j.0956-7976.2005.01548.x 15869701

[B3] Bassili J. N. (1979). Emotion recognition: the role of facial movement and the relative importance of upper and lower areas of the face. *J. Pers. Soc. Psychol.* 37 2049–2058. 10.1037/0022-3514.37.11.2049 521902

[B4] Beilock S. L. Kulp C. A. Holt L. E. Carr T. H. (2004). More on the fragility of performance: choking under pressure in mathematical problem solving. *J. Exp. Psychol. Gen.* 133 584–600. 10.1037/0096-3445.133.4.584 15584808

[B5] Bhushan B. (2015). “Study of facial micro-expressions in psychology,” in *Understanding Facial Expressions in Communication: Cross-Cultural and Multidisciplinary Perspectives*, eds Mandal M. K. Awasthi A. (Berlin: Springer), 265–286.

[B6] Bornemann B. Winkielman P. Van D. M. E. (2012). Can you feel what you do not see? using internal feedback to detect briefly presented emotional stimuli. *Int. J. Psychophysiol.* 85 116–124. 10.1016/j.ijpsycho.2011.04.007 21571012

[B7] Calder A. J. Young A. W. Keane J. Dean M. (2000). Configural information in facial expression perception. *J. Exp. Psychol. Hum. Percept. Perform.* 26 527–551. 10.1037/0096-1523.26.2.527 10811161

[B8] Calvo M. G. Fernandez-Martin A. Nummenmaa L. (2013a). A smile biases the recognition of eye expressions: configural projection from a salient mouth. *Exp. Psychol.* 66 1159–1181. 10.1080/17470218.2012.732586 23140405

[B9] Calvo M. G. Gutiérrezgarcía A. Avero P. Lundqvist D. (2013b). Attentional mechanisms in judging genuine and fake smiles: eye-movement patterns. *Emotion* 13 792–802. 10.1037/a0032317 23627721

[B10] Cohen J. (1988). *Statistical Power Analysis for the Behavioral Sciences*, 2nd Edn Hillsdale, NJ: Erlbaum.

[B11] Cui X. Wang Z. Jiang C. Tian B. (2009). Asymmetry of emotional information in upper and lower facial expression. *J. Psychol. Sci.* 32 1183–1185.

[B12] Davis J. I. Senghas A. Brandt F. Ochsner K. N. (2010). The effects of BOTOX injections on emotional experience. *Emotion* 10 433–440. 10.1037/a0018690 20515231 PMC2880828

[B13] Demetrioff S. Porter S. Baker A. (2017). I know how you feel: the influence of psychopathic traits on the ability to identify micro-expressions. *Psychol. Crime Law* 23 274–290. 10.1080/1068316x.2016.1247159

[B14] Dimberg U. Thunberg M. Elmehed K. (2000). Unconscious facial reactions to emotional facial expressions. *Psychol. Sci.* 11 86–89. 10.1111/1467-9280.00221 11228851

[B15] Ekman P. (1971). Universals and cultural differences in facial expressions of emotion. *Nebr. Symp. Motiv.* 19 207–283. 10.1037/0022-3514.53.4.712

[B16] Ekman P. (2002). *Micro Expression Training Tool.* San Francisco, CA: University of California.

[B17] Ekman P. (2003). Darwin, deception, and facial expression. *Ann. N. Y. Acad. Sci.* 1000 205–221. 10.1196/annals.1280.010 14766633

[B18] Ekman P. (2009). “Lie catching and microexpressions,” in *The Philosophy of Deception*, ed. Martin C. (New York, NY: Oxford University Press), 118–133. 10.1093/acprof:oso/9780195327939.003.0008

[B19] Ekman P. Friesen W. V. (1969). Nonverbal leakage and clues to deception. *Psychiatry* 32 88–97. 10.1080/00332747.1969.11023575 5779090

[B20] Ekman P. O’Sullivan M. (2006). From flawed self-assessment to blatant whoppers: the utility of voluntary and involuntary behavior in detecting deception. *Behav. Sci. Law* 24 673–686. 10.1002/bsl.729 17016820

[B21] Endres J. Laidlaw A. (2009). Micro-expression recognition training in medical students: a pilot study. *BMC Med. Educ.* 9:47. 10.1186/1472-6920-9-47 19619307 PMC2718872

[B22] Faul F. Erdfelder E. Buchner A. Lang A. G. (2009). Statistical power analyses using g^∗^power 3.1: tests for correlation and regression analyses. *Behav. Res. Methods* 41 1149–1160. 10.3758/brm.41.4.1149 19897823

[B23] Frank M. G. Herbasz M. Sinuk K. Keller A. Nolan C. (2009a). I see how you feel: training laypeople and professionals to recognize fleeting emotions. *Paper Presented at the Annual Meeting of the International Communication Association.* New York City, NY.

[B24] Frank M. G. Maccario C. J. Govindaraju V. (2009b). “Behavior and security,” in *Protecting Airline Passengers in the Age of Terrorism*, ed. Seidenstat P. (Santa Barbara, CA: Greenwood Pub), 86–106.

[B25] Frank M. G. Svetieva E. (2015). “Microexpressions and deception,” in *Understanding Facial Expressions in Communication: Cross-Cultural and Multidisciplinary Perspectives*, eds Mandal M. K. Awasthi A. (Berlin: Springer), 227–242.

[B26] Gandevia S. C. Burke D. (1992). Does the nervous system depend on kinesthetic information to control natural limb movements? *Behav. Brain Sci.* 15 614–632. 10.1017/cbo9780511529788.003

[B27] Hall J. A. Matsumoto D. (2004). Gender differences in judgments of multiple emotions from facial expressions. *Emotion* 4 201–206. 10.1037/1528-3542.4.2.201 15222856

[B28] Havas D. Glenberg A. Gutowski K. Lucarelli M. Davidson R. (2010). Cosmetic use of botulinum toxin-A affects processing of emotional language. *Psychol. Sci.* 21 895–900. 10.1177/0956797610374742 20548056 PMC3070188

[B29] Hawk S. T. Fischer A. H. Van Kleef G. A. (2012). Face the noise: embodied responses to nonverbal vocalizations of discrete emotions. *J. Pers. Soc. Psychol.* 102 796. 10.1037/a0026234 22059840

[B30] Hess U. Fischer A. (2014). Emotional mimicry: why and when we mimic emotions. *Soc. Pers. Psychol. Compass.* 8 45–57. 10.1111/spc3.12083 26876363

[B31] Hurley C. M. (2012). Do you see what i see? learning to detect micro expressions of emotion. *Motiv. Emot.* 36 371–381. 10.1007/s11031-011-9257-2

[B32] Hurley C. M. Anker A. E. Frank M. G. Matsumoto D. Hwang H. C. (2014). Background factors predicting accuracy and improvement in micro expression recognition. *Motiv. Emot.* 38 700–714. 10.1007/s11031-014-9410-9

[B33] Hyniewska S. Sato W. (2015). Facial feedback affects valence judgments of dynamic and static emotional expressions. *Front. Psychol.* 6:291. 10.3389/fpsyg.2015.00291 25852608 PMC4362049

[B34] Ipser A. Cook R. (2015). Blocking facial mimicry reduces perceptual sensitivity for facial expressions. *J. Vis.* 15:1376 10.1167/15.12.1376

[B35] Iwasaki M. Noguchi Y. (2016). Hiding true emotions: micro-expressions in eyes retrospectively concealed by mouth movements. *Sci. Rep.* 6:22049. 10.1038/srep22049 26915796 PMC4768101

[B36] Kaiser J. Gcl D. (2017). The effect of facial feedback on the evaluation of statements describing everyday situations and the role of awareness. *Conscious Cogn.* 53 23–30. 10.1016/j.concog.2017.05.006 28609702

[B37] Kontsevich L. L. Tyler C. W. (2004). What makes mona lisa smile? *Vis. Res.* 44 1493–1498. 10.1016/j.visres.2003.11.027 15126060

[B38] Korb S. With S. Niedenthal P. Kaiser S. Grandjean D. (2014). The perception and mimicry of facial movements predict judgments of smile authenticity. *PLoS One* 9:e99194. 10.1371/journal.pone.0099194 24918939 PMC4053432

[B39] Künecke J. Hildebrandt A. Recio G. Sommer W. Wilhelm O. (2014). Facial EMG responses to emotional expressions are related to emotion perception ability. *PLoS One* 9:e84053. 10.1371/journal.pone.0084053 24489647 PMC3904816

[B40] Lobmaier J. S. Fischer M. H. (2015). Facial feedback affects perceived intensity but not quality of emotional expressions. *Brain Sci.* 5 357–368. 10.3390/brainsci5030357 26343732 PMC4588143

[B41] Marsh P. J. Melissa J. G. Tamara A. Russell J. M. Anthony H. Max C. (2010). Remediation of facial emotion recognition in Schizophrenia: functional predictors, generalizability, and durability. *Am. J. Psychiatr. Rehabil.* 13 143–170. 10.1080/154877610037570

[B42] Matsumoto D. Hwang H. S. (2011). Evidence for training the ability to read microexpressions of emotion. *Motiv. Emot.* 35 181–191. 10.1007/s11031-011-9212-2

[B43] Matsumoto D. Leroux J. Wilsoncohn C. Raroque J. Kooken K. Ekman P. (2000). A new test to measure emotion recognition ability: matsumoto and ekman’s japanese and caucasian brief affect recognition test (jacbart). *J. Nonverbal Behav.* 24 179–209. 10.1023/A:100666812

[B44] Mehu M. Mortillaro M. Bänziger T. Scherer K. R. (2011). Reliable facial muscle activation enhances recognizability and credibility of emotional expression. *Soc. Psychol. Pers. Sci.* 2 262–271. 10.1037/a0026717 22642350

[B45] Mortillaro M. Mehu M. Scherer K. R. (2011). Subtly different positive emotions can be distinguished by their facial expressions. *Soc. Psychol. Pers. Sci.* 2 262–271. 10.1177/1948550610389080

[B46] Mu X. Li X. Zhong H. Li H. (2017). Fixation patterns of Chinese participants while identifying facial expressions on Chinese faces. *Front. Psychol.* 8:581. 10.3389/fpsyg.2017.00581 28446896 PMC5388684

[B47] Neal D. T. Chartrand T. L. (2011). Embodied emotion perception: amplifying and dampening facial feedback modulates emotion perception accuracy. *Soc. Psychol. Personal. Sci.* 2 673–678. 10.1177/1948550611406138

[B48] Niedenthal P. M. Brauer M. (2012). Social functionality of human emotion. *Annu. Rev. Psychol.* 63 259–285. 10.1146/annurev.psych.121208.131605 22017377

[B49] Niedenthal P. M. Mermillod M. Maringer M. Hess U. (2010). The simulation of smiles (SIMS) model: embodied simulation and the meaning of facial expression. *Behav. Brain Sci.* 33 417–433. 10.1017/S0140525X10000865 21211115

[B50] Oberman L. M. Winkielman P. Ramachandran V. S. (2007). Face to face: blocking facial mimicry can selectively impair recognition of emotional expressions. *Soc. Neurosci.* 2 167–178. 10.1080/1747091070139194 18633815

[B51] Ponari M. Conson M. D’Amico N. P. Grossi D. Trojano L. (2012). Mapping correspondence between facial mimicry and emotion recognition in healthy subjects. *Emotion* 12 1398–1403. 10.1037/a0028588 22642357

[B52] Porter S. Brinke L. T. Wallace B. (2012). Secrets and lies: involuntary leakage in deceptive facial expressions as a function of emotional intensity. *J. Nonverbal Behav.* 36 23–37. 10.1007/s10919-011-0120-7

[B53] Porter S. ten Brinke L. (2008). Reading between the lies: Identifying concealed and falsified emotions in universal facial expressions. *Psychol. Sci.* 19 508–514. 10.1111/j.1467-9280.2008.02116.x 18466413

[B54] Price T. F. Harmon-Jones E. (2015). Embodied emotion: the influence of manipulated facial and bodily states on emotive responses. *Wiley Interdiscip. Rev. Cogn. Sci.* 6 461–473. 10.1002/wcs.1370 26401657

[B55] Prodan C. I. Orbelo D. M. Testa J. A. (2001). Hemispheric differences in recognizing upper and lower facial displays of emotion. *Cogn. Behav. Neurol.* 103 248–256. 11725213

[B56] Ross E. D. Reddy A. I. Nair A. Mikawa K. Prodan C. I. (2007). Facial expressions are more easily produced on the upper-lower compared to right-left hemiface. *Percept. Mot. Skills* 104 155–165. 10.2466/pms.104.1.155-165 17450976

[B57] Russell T. A. Chu E. Phillips M. L. (2006). A pilot study to investigate the effectiveness of emotion recognition remediation in schizoprenia using the micro-expression training tool. *Br. J. Clin. Psychol.* 45 579–583. 10.1348/014466505x90866 17076965

[B58] Russell T. A. Green M. J. Simpson I. Coltheart M. (2008). Remediation of facial emotion perception in schizophrenia: concomitant changes in visual attention. *Schizophr. Res.* 103 248–256. 10.1016/j.schres.2008.04.033 18565733

[B59] Rychlowska M. Cañadas E. Wood A. Krumhuber E. Fischer A. Niedenthal P. (2014). Blocking mimicry makes true and false smiles look the same. *PLoS One* 9:e90876. 10.1371/journal.pone.0090876 24670316 PMC3966726

[B60] Shen X. B. Wu Q. Fu X. (2012). Effects of the duration of expressions on the recognition of microexpressions. *J. Zhejiang. Univ. Sci. B* 13 221–230. 10.1631/jzus.b1100063 22374615 PMC3296074

[B61] Shen X. B. Wu Q. Zhao K. Fu X. (2016a). Electrophysiological evidence reveals differences between the recognition of micro-expressions and macro-expressions. *Front. Psychol.* 7:1346. 10.3389/fpsyg.2016.01346 27630610 PMC5005928

[B62] Shen X. B. Yan W. Fu X. (2016b). “Recognizing fleeting facial expressions with different viewpoints,” in *Proceedings of the International Conference on Fuzzy Systems and Knowledge Discovery*, (China: IEEE), 2565–2569. 10.1109/fskd.2015.7382360

[B63] Stel M. van Knippenberg K. A. (2008). The role of facial mimicry in the recognition of affect. *Psychol. Sci.* 19 984–985. 10.1111/j.1467-9280.2008.02188.x 19000207

[B64] Stewart P. A. Bucy E. P. Mehu M. (2015). Strengthening bonds and connecting with followers: a biobehavioral inventory of political smiles. *Politics Life Sci.* 34 73–92. 10.1017/pls.2015.5 26399947

[B65] Svetieva E. Frank M. G. (2016). Empathy, emotion dysregulation, and enhanced microexpression recognition ability. *Motiv. Emot.* 40 309–320. 10.1007/s11031-015-9528-4

[B66] Tamietto M. Castelli L. Vighetti S. Perozzo P. Geminiani G. Weiskrantz L. (2009). Unseen facial and bodily expressions trigger fast emotional reactions. *Proc. Natl. Acad. Sci. U.S.A.* 106 17661–17666. 10.1073/pnas.0908994106 19805044 PMC2764895

[B67] ten Brinke L. Porter S. (2012). Cry me a river: identifying the behavioral consequences of extremely high-stakes interpersonal deception. *Law Hum. Behav.* 36 469–477. 10.1037/h0093929 23205594

[B68] Warren G. Schertler E. Bull P. (2009). Detecting deception from emotional and unemotional cues. *J. Nonverbal Behav.* 33 59–69. 10.1007/s10919-008-0057-7

[B69] Weinberger S. (2010). Airport security: intent to deceive? *Nature* 465 412–415. 10.1038/465412a 20505706

[B70] Wingenbach T. S. H. Brosnan M. Pfaltz M. C. Plichta M. M. Ashwin C. (2018). Incongruence between observers’ and observed facial muscle activation reduces recognition of emotional facial expressions from video stimuli. *Front. Psychol.* 9:864. 10.3389/fpsyg.2018.00864 29928240 PMC5997820

[B71] Wojciechowski J. Stolarski M. Matthews G. (2014). Emotional intelligence and mismatching expressive and verbal messages: a contribution to detection of deception. *PLoS One* 9:e92570. 10.1371/journal.pone.0092570 24658500 PMC3962410

[B72] Wood A. Rychlowska M. Korb S. Niedenthal P. (2016a). Fashioning the face: sensorimotor simulation contributes to facial expression recognition. *Trends Cogn. Sci.* 20 227–240. 10.1016/j.tics.2015.12.010 26876363

[B73] Wood A. Lupyan G. Sherrin S. Niedenthal P. (2016b). Altering sensorimotor feedback disrupts visual discrimination of facial expressions. *Psychon. Bull. Rev.* 23 1150–1156. 10.3758/s13423-015-0974-5 26542827

[B74] Wu Q. Guo H. He L. (2016). Facial feedback and micro-expression recognition. *J. Psychol. Sci.* 39 1353–1358.

[B75] Yan W. J. Wang S. J. Liu Y. J. Wu Q. Fu X. (2014). For micro-expression recognition: database and suggestions. *Neurocomputing* 136 82–87. 10.1016/j.neucom.2014.01.029

[B76] Yan W. J. Wu Q. Liang J. Chen Y. H. Fu X. L. (2013). How fast are the leaked facial expressions: The duration of micro-expressions. *J. Nonverbal Behav.* 37 88–97. 10.1007/s10919-013-0159-8

[B77] Yin L. J. Wei X. Z. Sun Y. Wang J. Rosato M. J. (2006). “A 3D facial expression database for facial behavior research,” in *IEEE International Conference on Automatic Face and Gesture Recognition*, (Southampton: IEEE), 211–216. 10.1109/fgr.2006.6

[B78] Zhang M. Fu Q. Chen Y. H. Fu X. (2018). Emotional context modulates micro-expression processing as reflected in event-related potentials. *Psych J.* 7 13–24. 10.1002/pchj.196 29297992

[B79] Zhang M. Fu Q. F. Chen Y.-H. Fu X. (2014). Emotional context influences micro-expression recognition. *PLoS One* 9:e95018. 10.1371/journal.pone.0095018 24736491 PMC3988169

