# Supplementary material for: A False Trail to Follow: Differential Effects of the Facial Feedback Signals From the Upper and Lower Face on the Recognition of Micro-Expressions
Source: Front Psychol. 2018 Oct 24;9:2015. doi: 10.3389/fpsyg.2018.02015 (PMC6208096; doi:10.3389/fpsyg.2018.02015)
Supplement: Supplementary file 1 [file Data_Sheet_1.PDF]

Supplementary Material

A False Trail to Follow: Differential Effects of the Facial Feedback Signals from the Upper and Lower Face on the Recognition of Micro-Expressions

Xuemei Zeng, Qi Wu*, Siwei Zhang, Zheying Liu, Qing Zhou, Meishan Zhang

*** Correspondence:** Qi Wu: sandwich624@yeah.net

1 Results of Simple Effects Analysis for Ratings of Resistance in Study 1

Simple effects analysis showed that before applying the gel, the baselines of ratings of resistance of all regions in the three partial facial feedback groups were identical (*F*s < .32, *p*s > .72). After applying the gel, the main effect of region was significant for each partial facial feedback condition (*F*s > 859.74, *p*s < .001). As we expected, post-hoc comparisons[1] showed the ratings of resistance of the target areas in each partial facial feedback conditions were increased (See Supplementary Table 1).

Supplementary Table 1. The difference of ratings of resistance between different regions after facial feedback manipulation (Study 1)

	Upper face group			Lower face group			Control		
	Difference	*t*	*p*	Difference	*t*	*p*	Difference	*t*	*p*
Upper face - Lower face	5.3	36.51	*p* < .001	- 5.23	-36.05	*p* < .001	- $4.44\text{x}10^{-16}$	$-0.12\text{x}10^{-13}$	*p* > .99
Lower face - Arm	-5.32	-35.93	*p* < .001	-0 .02	-0.16	*p* > .99	- 5.32	35.93	*p* < .001
Upper face - Arm	-0 .02	0.16	*p* > .99	- 5.25	-36.71	*p* < .001	- 5.32	- 37.19	*p* < .001

The results also showed that, after applying the gel, the main effect of partial facial feedback was significant for the regions of upper face [$F(2, 129) = 756.44$, $p < .001$, $\eta_p^2 = .92$] and lower face [$F(2, 129) = 838.2$, $p < .001$, $\eta_p^2 = .93$], but not for the non-dominant inner arm [$F(2, 129) = 0.02$, $p = .99$, $\eta_p^2 < .001$]. Further post-hoc comparisons showed that the rating of resistance of upper face in the upper face group was significantly higher than those in the lower face and control groups, and the rating of resistance of lower face in the lower face group was significantly higher than those in the upper face and control groups (see Supplementary Table 2 & 3). The results also showed, after applying the gel, there were no significant differences in the ratings of resistance among the upper

[1] The Bonferroni test was employed for all post-hoc comparisons in the current study.

face in upper face group, the lower face in lower face group, and the arm in non-dominate inner arm group [$F(2, 129) = 0.02$, $p = .99$, $\eta_p^2 < .001$].

Supplementary Table 2. The difference of resistance between partial facial feedback groups after facial feedback manipulation in the upper face (Study 1)

Upper face	Difference	*t*	*p*
Upper face group - lower face group	5.21	33.37	$p < .001$
Lower face group - control	0.09	0.58	$p > .99$
Upper face group - control	5.30	33.94	$p < .001$

Supplementary Table 3. The difference of resistance between partial facial feedback groups after facial feedback manipulation in the lower face (Study 1)

Lower face	Difference	*t*	*p*
Upper face group - lower face group	-5.32	35.45	$p < .001$
Lower face group - control	5.32	33.45	$p < .001$
Upper face group - control	-1.78×10^{-15}	0.12×10^{-13}	$p > .99$

2 Results of Simple Effects Analysis for Ratings of Resistance in Study 2

Simple effects analysis showed that before applying the gel, the baselines of ratings of resistance of all regions in the three partial facial feedback groups were identical (*F*s < 0.76, *p*s > .47). After applying the gel, the main effect of region was significant for each partial facial feedback condition (*F*s > 708.07, *p*s < .001). As we expected, post-hoc comparisons showed the ratings of resistance of the target areas in each partial facial feedback conditions were increased (See Supplementary Table 4).

Supplementary Table 4. The difference of ratings of resistance between different regions after facial feedback manipulation (Study 2)

	Upper face group			Lower face group			Control		
	Difference	*t*	*p*	Difference	*t*	*p*	Difference	*T*	*p*
Upper face - Lower face	5.31	35.88	$p < .001$	- 5.39	-36.41	$p < .001$	-0.08	-0.56	$p > .99$
Lower face - Arm	-5.25	-40.04	$p < .001$	0.06	-0.37	$p > .99$	-5.19	-32.18	$p < .001$
Upper face - Arm	0.06	0.34	$p > .99$	- 5.33	-32.51	$p < .001$	-5.28	-34.40	$p < .001$

The results also showed that, after applying the gel, the main effect of partial facial feedback was significant for the regions of upper face [$F(2, 105) = 689.91$, $p < .001$, $\eta_p^2 = .93$] and lower face [$F(2, 105) = 752.18$, $p < .001$, $\eta_p^2 = .94$], but not for the non-dominant inner arm [$F(2, 105) = 0.13$, $p = .88$, $\eta_p^2 = .002$]. Further post-hoc comparisons showed that the rating of resistance of upper face in the upper face group was significantly higher than those in the lower face and control groups, and the rating of resistance of lower face in the lower face group was significantly higher than those in the upper face and control groups (see Supplementary Table 5 & 6). The results also showed, after applying the gel, there were no significant differences in the ratings of resistance among the upper face in upper face group, the lower face in lower face group, and the arm in non-dominate inner arm group [$F(2, 129) = 0.35$, $p = .71$, $\eta_p^2 = .007$].

Supplementary Table 5. The difference of resistance between partial facial feedback groups after facial feedback manipulation in the upper face (Study 2)

Upper face	Difference	*t*	*p*
Upper face group - lower face group	5.44	32.21	$p < .001$
Lower face group - control	-0.02	- 0.17	$p > .99$
Upper face group - control	5.42	32.05	$p < .001$

Supplementary Table 6. The difference of resistance between partial facial feedback groups after facial feedback manipulation in the lower face (Study 2)

Lower face	Difference	*t*	*p*
Upper face group - lower face group	-5.25	-33.44	$p < .001$
Lower face group - control	5.28	33.62	$p < .001$
Upper face group - control	0.02	0.17	$p > .99$
